# How Myalgic Encephalomyelitis/Chronic Fatigue Syndrome (ME/CFS) Progresses: The Natural History of ME/CFS

**DOI:** 10.3389/fneur.2020.00826

**Published:** 2020-08-11

**Authors:** Luis Nacul, Shennae O'Boyle, Luigi Palla, Flavio E. Nacul, Kathleen Mudie, Caroline C. Kingdon, Jacqueline M. Cliff, Taane G. Clark, Hazel M. Dockrell, Eliana M. Lacerda

**Affiliations:** ^1^Department of Clinical Research, Faculty of Infectious and Tropical Diseases, London School of Hygiene & Tropical Medicine, London, United Kingdom; ^2^B.C. Women's Hospital and Health Centre, Vancouver, BC, Canada; ^3^Department of Medical Statistics, Faculty of Infectious and Tropical Diseases, London School of Hygiene & Tropical Medicine, London, United Kingdom; ^4^Department of Global Health, School of Tropical Medicine and Global Health, Nagasaki University, Nagasaki, Japan; ^5^Pro-Cardiaco Hospital and Federal University of Rio de Janeiro, Rio de Janeiro, Brazil; ^6^Department of Infection Biology, Faculty of Infectious and Tropical Diseases, London School of Hygiene & Tropical Medicine, London, United Kingdom

**Keywords:** Myalgic Encephalomyelitis/Chronic Fatigue Syndrome, Chronic Fatigue Syndrome, ME/CFS, chronic illness, management, research

## Abstract

We propose a framework for understanding and interpreting the pathophysiology of Myalgic Encephalomyelitis/Chronic Fatigue Syndrome (ME/CFS) that considers wider determinants of health and long-term temporal variation in pathophysiological features and disease phenotype throughout the natural history of the disease. As in other chronic diseases, ME/CFS evolves through different stages, from asymptomatic predisposition, progressing to a prodromal stage, and then to symptomatic disease. Disease incidence depends on genetic makeup and environment factors, the exposure to singular or repeated insults, and the nature of the host response. In people who develop ME/CFS, normal homeostatic processes in response to adverse insults may be replaced by aberrant responses leading to dysfunctional states. Thus, the predominantly neuro-immune manifestations, underlined by a hyper-metabolic state, that characterize early disease, may be followed by various processes leading to multi-systemic abnormalities and related symptoms. This abnormal state and the effects of a range of mediators such as products of oxidative and nitrosamine stress, may lead to progressive cell and metabolic dysfunction culminating in a hypometabolic state with low energy production. These processes do not seem to happen uniformly; although a spiraling of progressive inter-related and self-sustaining abnormalities may ensue, reversion to states of milder abnormalities is possible if the host is able to restate responses to improve homeostatic equilibrium. With time variation in disease presentation, no single ME/CFS case description, set of diagnostic criteria, or molecular feature is currently representative of all patients at different disease stages. While acknowledging its limitations due to the incomplete research evidence, we suggest the proposed framework may support future research design and health care interventions for people with ME/CFS.

## Introduction

The lack of progress in Myalgic Encephalomyelitis/Chronic Fatigue Syndrome (ME/CFS) research has been attributed to a range of factors, including the paucity of large, high quality, hypothesis-driven studies, and controversy around diagnosis. Without recognized and validated biomarkers or diagnostic tests, there is an over-reliance on patient history for diagnosis, which is based on criteria with limited sensitivity and specificity (1) and which ignore disease sub-groups. Furthermore, the lack of consistency in the choice and application of research case definition has led to problems with reliability and comparability of research findings (2). An additional factor complicating diagnosis and case definition for research studies is the time-related variation in phenotype both in the short- (3, 4) and long-term (5), which has seldom been considered in research studies.

In addition to often marked variability in disease presentation, severity, progression, and duration among different individuals, the way disease manifests in each individual may change with time. Inter- and intra-individual phenotypic variations lend toward the categorization of different subtype trajectories of ME/CFS that may differ in pathogenesis and prognosis. In some studies, female sex, increased age (6–8), and lower socio-economic status (9) have been found to predict poor prognosis; however, the variable nature of both population sampling and diagnostic criteria has led to ambiguous results and has reinforced the need for ongoing research in this area (10). Further subtypes have been defined on the basis of “minor” symptoms i.e., musculoskeletal, infectious, or neurological (11), through genetic studies (12, 13), metabolomics studies (14), and, duration of disease studies (5), highlighting the multitude of possible ways ME/CFS patients can be categorized. Other studies have identified variations in symptom profiles as disease progresses; however, such results are often limited by cross-sectional study design (15), and/or recall bias (16). The breadth of subtype studies available follow a similar model of looking for patterns across patient groups at single time-points; far fewer consider longitudinal subtyping and disease progression of a single patient cohort over time.

The concept of the natural history of disease is well-understood in public health and medicine: many, if not all, diseases are framed using this construct to formulate how they progress from a pre-illness stage to a final disease outcome, which may vary from full recovery to death. A good understanding of the disease course is vital not only for the design of preventative and intervention studies (17), but also to assess the timing and type of intervention that minimizes disease risk or optimizes prognosis. Although there is some understanding of the natural history of ME/CFS, this has been limited by problems in case definition (as above) as well as by the paucity of longitudinal studies, and in particular those that follow up individuals' pre-illness. A review of studies on CFS prognosis (8) suggested recovery rates under 10% in adults, and an improvement rate over 40% for people with fatigue lasting <6 months. The prognosis was worse: when more stringent case definitions were used; in older people; in cases with more severe symptoms; and, in the presence of psychiatric co-morbidity. A subsequent systematic review on prognosis found a median recovery rate of 5%, and median proportion of people improving of 39.5% (18) with most reporting symptoms still present at follow-up.

This conceptual paper explores the long-term course of ME/CFS and how presentation and pathophysiological abnormalities may vary with time. The pathophysiological concepts discussed are based on evidence from clinical observations and research, where available, and, as such, are not claimed to be original or indeed conclusive. Instead, they serve to highlight our proposed characterization of ME/CFS's distinct stages within the framework of the natural history of the disease.

## Pathophysiological and Cellular Abnormalities Following Host Exposure to “Insults” or “Stressors”

Prior to exploring the course of ME/CFS, we propose to revisit some concepts related to mechanisms of disease that have been used in the context of life-threatening emergencies and to potential return to homeostasis, such as those occurring in sepsis or poly-trauma. Although very different to ME/CFS, these acute injuries have been extensively studied, and the high intensity and speed of events result in changes that are easily identified and well-described, from potential homeostatic failure to recovery. We present the following models as a paradigm for the understanding of disease mechanisms, based on well-studied examples. They merely serve as a reference for mechanisms that the host may partially engage with in the presence of insults of different severities. Hence, in the following paragraphs, we explore the pathophysiological mechanisms that may be taking place in ME/CFS, which have been related to abnormal homeostasis guided by these established disease descriptions.

The response to an insult frequently involves multiple body-systems and has components that are independent of the etiology of the insult and, to some extent, its severity. There are many commonalities between the response to sepsis and to poly-trauma: both are acute and severe insults, to which many of the aspects of the host response are indistinguishable. Our proposal is based on the idea that there may be some similar mechanisms at play when individuals predisposed to ME/CFS are faced with a range of “insults” or “stressors.” Needless to say, the hyper-acute changes and co-factors in both sepsis and poly-trauma occur in very rapid sequence, whereas in ME/CFS, physiological changes, even if they resemble those of acute injury in some respects, take place at a much slower pace with less obvious and uniform patterns.

### Non-specific Changes in Response to Severe Acute Injury

In both sepsis (19) and poly-trauma, (20, 21) a state of hyper-inflammation is observed initially as the host responds to the infection or traumatic stress with marked production of pro-inflammatory mediators, e.g., cytokines and polypeptides. A failing circulatory system is associated with activation of the hypothalamic-pituitary-adrenal (HPA) axis and increased sympathetic drive, contributing to metabolic changes and to increased energy expenditure (22, 23).

In these conditions, the acute pro-inflammatory state is usually followed by a compensatory anti-inflammatory response, with a different profile of biochemical and molecular mediators. The success of the host in balancing pro- with anti- inflammatory responses alongside injury-related factors, are key to improved long-term outcomes. The direct and indirect effects of immune cells and active products derived from immune, neural, and endocrine systems (some of which cause pathology if present in excess) contribute to a number of physiological changes, including those leading to the formation of reactive oxygen species (ROS, oxidative stress) and reactive nitrogen species (RNS, nitrosative stress). Endothelial and parenchymal (organ) cell damage may result because of a combination of factors, such as polymorphonuclear leukocyte infiltration and the action of reactive oxygen and nitrogen species, cytokines, vasoactive amines, and other products. Endothelial dysfunction results in capillary leakage, accelerated inflammation, platelet aggregation, coagulation, and loss of vascular tone (24). Vascular dysfunction is associated to peripheral vasodilation due to increased nitric oxide and prostacyclin synthesis (25) and to a decrease in the proportion of perfused vessels and an increase in the heterogeneity of blood flow distribution (26). This results in relative hypovolemia, decreased capillary flow, haemo-concentration, and micro-thrombi formation, and further contributes to reduced exchanges of oxygen and nutrients at the microcirculatory level. The consequent decreased cellular oxygen delivery eventually leads to cytopathic hypoxia. Adenosine triphosphate (ATP) increased consumption and ensuing deficits cascade into a range of metabolic disturbances with systemic effects (27), and promote changes in membrane permeability that lead to dysfunctional transmembrane ion transport. In acutely and severely ill patients, reperfusion results in further oxidative damage (22, 28). Additional failures of biological and cell processes lead to multiple dysfunction, to system and organ failure, and to potentially irreversible disease (22).

### Evidence of Abnormalities in ME/CFS and Loss of Normal Homeostasis

Concepts that are relevant here are those of homeostasis and allostasis. While homeostasis refers to the “*stability of physiological systems*,” allostasis has been defined as “*the adaptive processes aimed to maintain homeostasis following acute stress, and which contribute to wear and tear on the body and the brain, or allostatic overload*” (29). A central characteristic of individuals with ME/CFS points to a state of homeostatic failure (30), aggravated by the incidence of, or increase in, levels of new stressors or by the increase in allostatic load (31). Typical stressors include infection [(32): 17–21], physical exertion and cognitive effort (e.g., reading or solving mental puzzles) triggering post-exertional malaise (PEM) (33), comorbid conditions (e.g., sleep disturbances) (34) and a range of environmental and individual factors (35–40).

In those who do not develop ME/CFS or prolonged illness following an insult such as an acute infection, external stressors may initially cause physiological changes accompanied by non-specific symptoms, but the state of homeostatic equilibrium that operated before the insult is quickly restored. Failing re-establishment of this equilibrium, there may be a shift to a state of “aberrant homeostasis,” where physiological processes converge to a new or alternative state of functioning; a state that remains homeostatic in nature, but functions at a less optimum level (41). While such a state may be adequate for many physiological processes, it will be inadequate or inefficient for a number of other processes and functions and the prolongation of such aberrant functioning will represent another potential source of ongoing stress.

There is a growing body of evidence on biological abnormalities in ME/CFS that has been reviewed elsewhere (32, 42, 43), and summarized by Komaroff (44). Of note, many of the abnormalities shown in severe injury have also been identified in ME/CFS such as: immune dysfunction, including pro-inflammatory response (especially at early stages of disease) (45, 46); autonomic nervous system (47–49); HPA axis dysfunction (50); hypovolemia (51); nitrosamine and oxidative stress (52); endothelial dysfunction (52); metabolic dysfunction (53–55); dysfunction of membrane transport (56); and, tissue hypoxia (57).

## The Stages of ME/CFS

Other tools widely used in clinical medicine are staging systems. Using sepsis again as an example, such a system was proposed at the International Sepsis Definitions Conference in 2001 to introduce the stratification of patients with sepsis (58). By applying PIRO (predisposition, infection/insult, response, and organ dysfunction) patients are stratified into appropriate subgroups allowing for more accurate prognostication in emergency medical services (59). The idea of classifying people with ME or CFS into distinct categories or stages has been explored previously by several theorists. One school of thought proposes categories based on the psychological process of coming to terms with this new and evolving state of health rather than addressing biological differences, and are defined as such by the emotions common to any trauma experience: e.g., denial, fear, frustration, and acceptance (60, 61). Alternatively, Schweitzer (62) proposes the different presentations of CFS according to more physical categories (Prodrome, Relapse and Remission, Improvement and Plateau, and Collapse followed by slow worsening with no remission); it is these that we aim to expand on, as follows.

**Figure 1:**
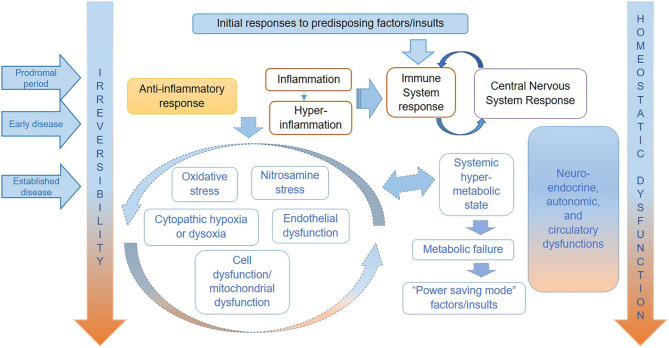
Hypothesized key pathophysiological mechanisms for ME/CFS.

We show a tentative representation of the key pathophysiological mechanisms operating in each stage of ME/CFS in Figure 1. As in severe injury or sepsis, the range and order of occurrence of biological processes taking place in ME/CFS may vary, as may their relative significance and impact on each individual. Therefore, it is important to note that although the various abnormalities may occur continuously and often simultaneously, the predominance of specific dysfunctions varies over time and from individual to individual.

Furthermore, we propose a characterization of disease stages in ME/CFS, based on the natural history of disease framework considering available descriptions from the literature (32), and the life-stories reported by our own cohort of research participants with ME/CFS (including those with mild/moderate or severe symptoms) (63). This characterization is summarized in Table 1, which may be used in support of research designs that consider the disease presentation in distinct phases.

**Table 1:** Proposed characterization of disease stages in an individual with ME/CFS, within the framework of natural history of diseases.

**Timing**	**No disease**	**Onset**	**0–4 months^**¶**^**	4–24 months^*^	**2 years +^†^**
**Stage**	**Predisposition**	**Trigger and pre-illness**	**Prodromal period**	**Early disease**	**Established disease**
Clinical phenotype	No symptoms	Non-specific or related to triggering “insult”	Fatigue-complex symptoms^‡^	Fatigue-complex symptoms^‡^ variable severity and progress	Mild, moderate, severe and complicated disease
Prevention level^¶^	Primary prevention	Treatment of “insult” and primary prevention	Secondary prevention	Treatment and secondary prevention	Treatment and tertiary prevention
Recovery Potential^§^			Likely	Possible	Less likely
Pathophysiology	Predisposing factors	Non-specific host response and related to specific trigger factor	Neuro-immune response to insult and fight for homeostasis	Neuro-inflammation and systemic consequences; aberrant homeostasis	Systemic disease, aberrant or failed homeostasis

* *3–6 months is commonly proposed as the minimum period of symptoms before diagnosis is made in children and adults, respectively (32)*.

*2 years has been used as a cut off to distinguish between short and long term duration of disease (94, 95), but its use as defining established disease is variable and depends on a range of factors, including individual response to early disease*.

‡ *Fatigue-complex symptoms: initially predominantly neuro-immune (prior to early disease), and variable systemic symptoms in established disease*.

§ *Tentative proportions for recovery are: likely (>75%); possible (<20%); less likely (<5%). “Likely” and “possible” are based on recovery from arboviruses and EBV [(96); 100]; “less likely” is based on reviews on prognosis (97)*.

¶ *The Prevention level will be considered further in a subsequent publication which is being prepared by the authors*.

### Predisposition and Triggering of Disease

Individuals with a combination of genetic predispositions and exposures to environmental factors may first manifest symptoms of ME/CFS following their encounter with a specific trigger, of which acute infections of various etiologies are the most commonly reported (64, 65); other patients report a more insidious onset with no obvious initiating factor (32). While it remains unclear exactly which individuals are predisposed to develop ME/CFS and why, some patterns have emerged. For example, gender- and age-specific factors are thought to contribute to the risk of ME/CFS (66), with epidemiological studies consistently reporting higher rates of the disease in females (67, 68). Although most cases are endemic, there have been reports of epidemic cases, suggesting an infectious or other environmental cause play a role (43, 69–72); although discrepancies in onset patterns and case definitions make these epidemics difficult to compare (72). Many studies have reported an association between acute viral infection and the development of ME/CFS (73–76). Cases are predominantly reported in North America, Europe, and Oceania; however, the occurrence of ME/CFS is thought to be global with evidence of cases in other parts of the world (77–79).

Psychiatric morbidity, experiences of stress and trauma, either physical or emotional have been reported to precipitate the disease (16, 80–82) and to predict disease progression (83), under the explanatory biopsychosocial models. However, these models have not been replicated (84, 85). Furthermore, Chu et al. (16) found that even when a significant proportion of their research population report stress or a major life event as a precipitating factor for ME/CFS, “*stressful events were rarely chosen as the only precipitant though, endorsed only by 8% of our subjects, and appeared mostly in conjunction with infection or other precipitants.”* We acknowledge that stress may play a role in the development and perpetuation of ME/CFS through its role on the immune system and HPA axis dysfunction (86), or by aiding transmission or reactivation of viral infections (87), or as a consequence of the loss of normal functioning experienced by the individual.

The role of genetic variation has been supported by a number of family-based studies assessing the possibility of a heritable component (88–90). Genes underpinning immune system function and inflammatory response may contribute to genetic susceptibility for ME/CFS; some studies suggest associations with human leucocyte antigen class II alleles (91, 92) and in genes related to the complement cascade, chemokines, cytokine signaling, and toll-like receptor signaling (93). Small genome-wide association studies (GWAS) have had little overlap in results save for two SNPs in the GRIK2 gene: a gene implicated in a number of neurological conditions such as autism and schizophrenia (98); in the GRIK3 gene: relating to a pattern recognition receptor capable of binding to a broad range of pathogens; and in the non-coding regions of T-cell receptor loci (99). A further study reported SNP markers in candidate genes involved in HPA axis function and neurotransmitter systems that distinguished individuals with ME/CFS (100).

### Prodromal Period

It is important to preface here that, with the current diagnostic methodology of ME/CFS stipulating the presence of symptoms for more than 6 months (101, 102) and the absence of a positive validated diagnostic test, the following processes (occurring pre-diagnosis) are difficult to substantiate from existing biomedical research. However, based on the published work on ME/CFS and considering the pathophysiological events happening in sepsis and polytrauma may be similar (though in a much slower pace), we hypothesize that the following may occur.

In addition to any manifestations specifically related to the acute insult or triggering event, the mechanisms involved in producing the first symptoms of ME/CFS may be similar to what has been described in relation to “sickness behavior” (103) or in those with severe acute disease, i.e., “systemic inflammatory response syndrome” (19). These result from the interaction of an infective agent or other insult with the host's immune system, as well as their potential effect on the host's central nervous system (CNS). The immune system-nervous system interactions involve bidirectional signals (104–106): while immune system activity may interfere with CNS function via various mechanisms, e.g., release and action of pro-inflammatory cytokines and other mediators, various neurotransmitters, neuropeptides, and neuro-hormones may also affect immune function. Additionally, the HPA system and the autonomic nervous system (ANS) are affected, with consequences that may be observed well-beyond the CNS. These effects may vary according to different factors, such as host susceptibility, the nature and persistence (or return to normality) of systemic and local immune dysfunction, altered CNS metabolism, neuro-transmission, brain perfusion changes, and the integrity of the blood-brain barrier (107–110).

Particular characteristics of the specific infectious agent or stressor may also play a role during this prodromal stage, which would explain the different risks of disease development following acute infection. For example, there has long been an interest in the association between ME/CFS and infections such as Epstein-Barr virus (EBV) and other herpesviruses (73, 111–116). Herpesviruses tend to be neurotropic and persist following acute infection in a latent state. Similar to EBV infection (117), the risk of chronic fatigue has been shown to be substantially increased following viral meningitis, a relatively severe infection of the CNS (83).

### Early Disease

Early disease represents a continuation of the processes initiated at the prodromal period, when there is a failure of physiological and homeostatic processes to resume previous levels of equilibrium and normality. Fatigue and other symptoms may be largely explained by a combination of the local and systemic effects of pro-inflammatory and other mediators or toxins, CNS metabolic dysfunction (with enhanced excitability and other changes), and a systemic hyper-metabolic state. With higher energy demands for essential biological processes, there will be a reduction in the available energy for less essential tasks, including those demanding increased physical or mental exertion. The increased production and action of anti-inflammatory mediators, as well as their ability to counter-balance pro-inflammatory stimuli, modulate physiological responses, and symptoms and affect disease progression or reversibility. As mentioned previously, without a validated biomarker to diagnose ME/CFS early it is difficult to substantiate the exact mechanisms occurring in the early disease phase. Research into potential diagnostic markers, such as the recent study on impedance signatures (118), are crucial not only clinically, but to identify these mechanisms as possible targets for early intervention.

### Established ME/CFS

The persistence of immune and CNS dysfunction with the initial over-production of pro-inflammatory and neurotoxic factors may result in a prolonged state of low-grade neurological and systemic inflammation. In the CNS, a status of glial activation with microglial hypersensitivity to peripheral (119) and regional stimuli is established (104, 119–121), akin to what has been described in chronic pain states (122). In support of CNS dysfunction, neuroimaging studies have shown various abnormalities in ME/CFS, often associated with symptoms of fatigue and other indications of severity (123). Glial activation in several areas of the brain has also been demonstrated in positron emission tomography (PET) scans of patients with fibromyalgia (FM), compared to controls, which was correlated to the severity of fatigue (123, 124). Neuro-glial bidirectional signaling is associated with increased production of neuro-excitatory neurotransmitters and immune-inflammatory mediators (120).

Nervous system dysfunction affecting parts of the brain, brain stem, and ANS, could explain not only the encephalopathic or neuro-cognitive type of symptoms, but also those resulting from disruption of key central regulatory mechanisms, such as those involved in endocrine, circulatory, thermoregulation, and respiratory control (16, 32, 48, 120, 125). Examples of these include intolerance to extremes of temperature, chills and temperature variations, intolerance to exertion, hyperventilation or irregular breathing, orthostatic intolerance, with hypotension or postural orthostatic tachycardia, and other symptoms related to autonomic and endocrine control function (102).

Among the various by-products produced as a consequence of ongoing abnormalities, are highly ROS and nitric oxide synthase (NOS) or free radicals, which affect cell signaling and cell functioning and structure, particularly when present at high levels. It has been hypothesized that free radicals, and increased levels of nitric oxide and peroxynitrite in particular, play a significant role in ME/CFS (126, 127); their links to immune and neuro signaling, cell integrity, mitochondrial function, and energy metabolism may play an important part in the long term abnormalities in ME/CFS.

The nature of neuro-immune and other dysfunctions may change as disease progresses. While a pro-inflammatory state is typical of the early response to insults, immune abnormalities may become less marked (and less pro-inflammatory) with time (128), and patients with longer periods of illness may show fewer inflammatory immunological abnormalities. In support of this, our preliminary results from the analysis of over 200 ME/CFS patients participating in the UK ME/CFS Biobank (UKMEB), showed that the reported time since disease onset was significantly associated with 2 cytokines, namely SCD40L and IL1RA (manuscript in preparation). These results were found after aliquots of peripheral blood mononuclear cells (PBMC) from participants were stimulated (i.e., subjected to an infection resembling stimulus) and analyzed with MAGPIX® multiplexing system. The statistical analyses were conducted after transforming each cytokine measurement to the logarithm scale to approximate normality; linear regression of these log-transformed values (adapted for truncated outcome variables to account for the assay's limits of detections) was applied to the variables' time since onset, level of severity (mild to moderate vs. severe) and the interaction between severity and time since onset, while also adjusting for age and sex. The results evidenced a decrease of sCD40L—a pro-inflammatory cytokine—and an increase of IL1RA—an anti-inflammatory cytokine—for every additional year since onset of ME.

### Long-Term, Advanced, and Complicated Disease

As the disease progresses, physiological, and systems abnormalities take their toll and cell dysfunction becomes more pronounced. Endothelial dysfunction may arise as a consequence of a range of factors, including, but not limited to, persistent oxidative and nitrosative stress and circulatory dysfunction (43, 52, 126, 129, 130). The associated reduced delivery of oxygen and nutrients to the cell leads to a deterioration of cell function and impaired energy metabolism (129, 131, 132) and a decreased ability of the cell to extract oxygen and produce energy, a condition known as cytopathic hypoxia. As suggested by Naviaux et al. (54), in cases of ME/CFS with mean duration of symptoms over 17 years, there is a shutting down of various metabolic processes leading to a hypometabolic state, i.e., a move to an energy-saving mode. At this stage, symptoms are likely to be severe, with profound fatigue, intolerance to effort, PEM and other systemic symptoms, which are largely explained by the slowness of physiological and metabolic processes and decreased energy production.

## Discussion

### Disease Severity and Reversibility

It is unknown how the initial host response to a stressor or insult compares in individuals who do or do not develop typical symptoms of ME/CFS. However, the return to good health, which happens to most people following exposure to mild or moderate levels of insult, seems to be impeded in ME/CFS when symptoms persist for longer than 3–6 months; the time interval that is featured in some of the currently used diagnostic criteria (2, 101, 102). This suggests that subsequent mechanisms involved in the host response will differ at some point in those who develop ME/CFS from those who regain full health. Therefore, a key question is what determines full recovery? Or alternatively, what determines the perpetuation and transformation of symptoms?

While the abnormalities observed in acute disease are general and mostly reversible once the challenge from the stressor ceases, some degree of dysfunction may persist for longer periods. The degree of reversibility of various physiological abnormalities is likely to decrease with time, and some permanent functional, and even structural, damage may occur consequently. This is likely caused by either the persistence or frequent reactivation of the initial stressor (87, 133), an accumulation of insults, a continuing dysfunctional host-response, or the effects of the numerous psychosocial risk factors that influence disease development and progression (134), or a combination of all of these.

Although our framework focuses on the underlying biological mechanisms that may be at play in the development and progression of ME/CFS, it is important to acknowledge the impact of psychosocial and behavioral aspects in the progression of chronic diseases. Stressors such as stressful life events, low satisfaction with social and medical support, and excessive use of coping mechanisms, have been shown to contribute to the neuroendocrine and immune responses by acting through complex pathways that ultimately affect health and health outcomes (134–136).

The interplay between these three dimensions (biological, psychosocial, and behavior) has been noted in the development and the progression of a number of chronic diseases and to influence disease outcomes (136–139). The combined effects of stress from work or family life, social deprivation, and depression have been found to contribute to the risk of cardiovascular diseases, including coronary heart disease (140) and myocardial infarction (141), and to a worse prognosis (142) by enhancing cortisol secretion, increasing sympathetic activation, and elevating plasma catecholamine levels (143). A higher cumulative average number of stressful life events, when coping involves denial, and higher levels of serum cortisol have been found to be associated with a faster progression to AIDS (144). Correspondingly, low stress levels and low scores of avoidance coping behaviors were shown to be protective against relapse in Crohn's disease patients (135) in contrast to high levels, which act as mediators, overloading the sympathetic nervous system.

In the case of ME/CFS, the effect of these dimensions is the same. In fact, one framework has been used to propose a model for managing patients with this disease in which it is considered that genes predispose, life events precipitate, and behaviors perpetuate (145–147). However, this model may downplay the important role of the biological mechanisms involved in ME/CFS and overstate the role of psychosocial and behavioral factors (148).

The pathophysiological distinction between cases from the milder to the more severe end of the ME/CFS spectrum may relate to near-normal homeostatic regulation in milder cases, and established “aberrant homeostasis” or homeostatic dysregulation with multi-systemic consequences in moderate to severe cases. Alternatively, homeostatic failure, along with variable multi-system physiological failure and increasing degrees of irreversibility, may happen in the most severe cases.

The early stage of ME/CFS is of variable duration but is usually considered to be between 4 and 6 months to 2 years after the start of prodromal symptoms. Reversibility is possible, but often people will evolve to chronicity or established ME/CFS with either: (a) partial reversal of dysfunctional physiological mechanisms (mild cases with slow improvement over time); (b) persistence of dysfunctions and symptoms (mild or moderate cases with stable symptoms or slow changes over time); or (c) worsening dysfunctions and symptoms (moderate and severe cases) (149). Note that some cases present early with severe symptoms, which not uncommonly evolve to a milder form (150). The use of coping mechanisms, such as pacing, can also help improve energy management in people with ME/CFS over time and reduce the risk of relapse into a more severe state; however, there is little evidence that these will lead to a reversibility (151). There is some indication that rates of resolution are higher in cases of epidemic CFS compared to sporadic cases, although very few of these individuals will recover to their pre-morbid level (152).

**Figure 2:**
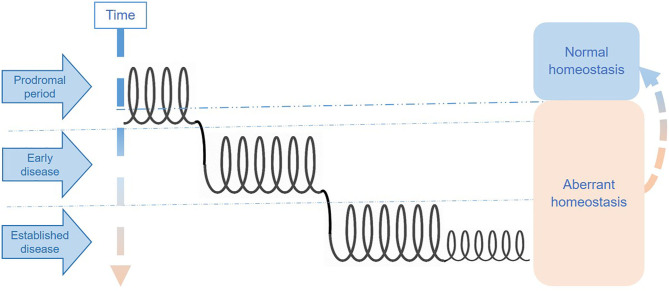
Hypothetical stages of disease in ME/CFS.

One way of thinking about these phases is as interconnected spirals, each representing a distinct disease phase. Individuals may either remain for long periods in a single phase with symptoms fluctuating within the “spiral section” or move between phases either upwards (i.e., toward better health status) or downwards (i.e., toward disease deterioration). Figure 2 represents an illustration of the multi-spiraling disease course suggested for ME/CFS, and shows how patients may move across spirals, with different molecular and system abnormalities.

### Common Comorbidities in ME/CFS

There are a number of comorbid conditions associated with ME/CFS and, as such, these comorbidities can complicate diagnosis, treatment and research of the disease. Comorbidities have been found in up to 97% of people with ME/CFS (PWME) (16, 153) with some developing before, with, or after ME/CFS onset (102). The complexity of ME/CFS is in part due to the number of different systems affected that contribute to the many and varied symptoms experienced. ME/CFS and FM share a number of overlapping core symptoms that mean the two are commonly experienced together; FM has been reported to co-occur in 12–91% of PWME (16, 154, 155). However, there is evidence to suggest the two conditions differ in their hormone dynamics, genetic/molecular biology, and autonomic function (156, 157). This is reiterated by the absence of post-exertional malaise in FM (158, 159), which is one of the key features of ME/CFS (2, 101, 102, 160).

Sleep disturbances can cause some symptoms that are also present in ME/CFS including fatigue, joint pain, and impaired cognition (161–165). Additionally, as part of a bidirectional relationship, comorbid pain conditions may further impact sleep quality (34). Sleep disturbances are also present in a number of neurological diseases (166), which would explain their presence as an important feature in ME/CFS (2, 160); however, differences in sleep cycle patterns and distinct sleep phenotypes suggest that ME/CFS and primary sleep disorders are, in fact, different entities (167, 168) with many PWME showing normal sleep study results (169). Primary sleep disturbances are considered exclusionary for ME/CFS by a number of diagnostic criteria (101, 102, 160), however, with little evidence that treatment of these disorders improves symptoms of ME/CFS it is argued they are better considered as comorbid conditions (2, 34, 170).

Also highly prevalent in those with ME/CFS is orthostatic intolerance (OI), a common multifactorial disorder commonly accompanying neurodegenerative, cardiovascular, metabolic, and renal disorders (171). Disruptions to ANS and reduced blood volume contribute to OI (172) and the same systemic dysfunctions have been reported in those with ME/CFS (51); however, not all people with OI disorders have ME/CFS (173, 174).

Intestinal dysbiosis thought to be associated with some CNS-related disorders via the gut-brain-axis (175). IBS is another largely overlapping syndrome with both ME/CFS and FM but metabolic profiles are distinct in ME/CFS and ME/CFS with IBS subgroups (176). Some authors hypothesize IBS could be considered an initial symptom of ME/CFS, as they reported that 65% of IBS patients followed up developed ME (177). Authors of a co-twin control study found significant associations between CFS and FM, IBS, chronic pelvic pain, multiple chemical sensitivities, and temporomandibular disorder. After controlling for psychiatric risk factors, they argued that these associations could not be attributed to uniquely psychiatric illness, thus suggesting a “*complex interplay of genes and environmental factors”* to help explain the clinical picture (178).

While healthcare costs likely increase following the diagnosis of additional comorbidities (178), treating comorbidities may improve the quality of life of PWME (2) not only symptomatically but also in what they might be able to contribute to the economy. We argue that by using the proposed natural history framework, how and when common comorbidities develop in relation to ME/CFS may be highlighted, allowing researchers, and clinicians to better tailor potential interventions according to each phase, thus resulting in a more efficient management of costs.

### Research Implications

These distinct hypothetical stages may help explain the apparent inconsistency of findings from ME/CFS studies, which likely include cases at distinct stages of disease with potentially diverse systems abnormalities. Hence, we consider that the conceptual approach presented in this paper may help to elucidate pathophysiological mechanisms that may be more prominent at different stages of disease; and consequently, could indicate potential target therapeutic approaches in future. We argue that the different stages patients go through during the course of the disease, their severity, and the presence and degree of complications are key parameters for disease stratification.

Research leading to an understanding of what is occurring during the first three stages of progression to ME/CFS is greatly needed but requires the recruitment of individuals for research at pre-illness stage. Such research could be invaluable to understanding the biological mechanisms at play before, during and after an insult, and research using proxy disease models for ME/CFS (85) or follow up of patients after an acute viral infection [e.g., mononucleosis (76) or more presently COVID-19] could begin to address this knowledge gap. Electronic health records could also be a valuable source of retrospective pre-illness data in people with ME/CFS. Well-designed longitudinal studies, with strict protocols, would help refine this attempted description of the natural course of the ME/CFS, and the interpretation of the findings.

## Conclusions

The concept of the natural history of disease, common in the field of public health and medicine, serves to frame a disease according to how it progresses from a pre-illness stage to the final disease outcome. Due to the lack of knowledge surrounding the etiology of ME/CFS, the heterogeneous presentation of symptoms and their severity, and the lack of a recognized and validated biomarker to determine diagnosis, the natural history of this disease has been hard to determine. While current research efforts tend to group ME/CFS subtypes according to clusters of symptoms, few studies have considered ME/CFS as a continuum.

Pathophysiological patterns and changes along and across disease stages result in the expression of different, albeit overlapping phenotypes as seen in the preliminary UKMEB findings related to changes in cytokine levels and symptoms scores with time of disease, reported here. Ignoring phenotype temporal variation in ME/CFS may have an impact on the outputs and the interpretation of research investigating disease mechanisms, pathways, and interventions.

This paper sought to provide a simple framework, similar to those of other chronic diseases, in an effort to extend the temporal perception of ME/CFS and better incorporate the less defined pre-illness stages of the disease. We believe that by applying this framework to ME/CFS research efforts could better elucidate the pathophysiological mechanisms of the disease and identify potential therapeutic targets at distinct stages.

## Data Availability Statement

The datasets generated for this study are available on request to the corresponding author.

## Ethics Statement

Ethical approval was granted by the LSHTM Ethics Committee 16 January 2012 (Ref.6123) and the National Research Ethics Service (NRES) London-Bloomsbury Research Ethics Committee 22 December 2011 (REC ref.11/10/1760, IRAS ID: 77765). All biobank participants provided written consent for questionnaire, clinical measurement and laboratory test data, and samples to be made available for ethically approved research, after receiving an extensive information sheet and consent form, which includes an option to withdraw from the study at any time and without any restrictions.

## Author Contributions

LN and EL conceived the paper. LP and EL provided the preliminary findings from data from the UKMEB participants and possible interpretation of them. SO'B contributed to drafting, referencing, and formatting. All authors contributed to drafting and to revising the manuscript and approved the final version to be published.

## Conflict of Interest

The authors declare that the research was conducted in the absence of any commercial or financial relationships that could be construed as a potential conflict of interest.
